# Proteomic Profiling of Endometrial Cancer Tissues Reveals Differential Expression of Proteomes in Obese Versus Non-Obese Patients

**DOI:** 10.3390/cells15060498

**Published:** 2026-03-11

**Authors:** Khalid Akkour, Mohamed Rafiullah, Assim A. Alfadda, Ibrahim O. Alanazi, Afshan Masood, Salini Scaria Joy, Ahood A. Al-Eidan, Eman Alshehri, Ali Bassi, Hani Alhalal, Amal AlQarni, Rukhsana Gul, Hicham Benabdelkamel

**Affiliations:** 1 Obstetrics and Gynecology Department, College of Medicine, King Saud University, Riyadh 11461, Saudi Arabia; kakkour@ksu.edu.sa (K.A.); emhalshehri@ksu.edu.sa (E.A.); abassi@ksu.edu.sa (A.B.); 2 Strategic Center for Diabetes Research, College of Medicine, King Saud University, Riyadh 11461, Saudi Arabia; mrafiullah@ksu.edu.sa (M.R.); aalfadda@ksu.edu.sa (A.A.A.); sjoy@ksu.edu.sa (S.S.J.); 3 Proteomics Resource Unit, Obesity Research Center, College of Medicine, King Saud University, Riyadh 11461, Saudi Arabia; afsmasood@ksu.edu.sa (A.M.); rgul@ksu.edu.sa (R.G.); 4 Department of Medicine, College of Medicine, King Saud University, Riyadh 11461, Saudi Arabia; 5 Healthy Aging Research Institute, King Abdulaziz City for Science and Technology (KACST), Riyadh 11442, Saudi Arabia; ialenazi@kacst.edu.sa; 6 Department of Biology, College of Science, Imam Abdulrahman Bin Faisal University, Dammam 34212, Saudi Arabia; aeidan@iau.edu.sa; 7 Obstetrics and Gynecology Department, King Saud University Medical City, King Saud University, Riyadh 11461, Saudi Arabia; halhalal@ksu.edu.sa (H.A.); amalaliqarnii@gmail.com (A.A.)

**Keywords:** endometrial cancer, obesity, proteomics, cancer metabolism

## Abstract

Endometrial cancer (EC) is the leading gynecological malignancy worldwide. Obesity is reported to be associated with 50% of EC cases. Significant gaps remain in investigating specific molecular mechanisms behind the obesity-driven EC. Women diagnosed with EC undergoing total hysterectomy were recruited. Patients were divided into two groups: EC-obese with BMI > 29.9 kg/m^2^ (*n* = 10) and EC-Non-obese with BMI ≤ 29.9 kg/m^2^ (*n* = 10). Tumor tissues were subjected to label-free quantitative proteomic analysis using liquid chromatography-tandem mass spectrometry (LC-MS/MS). Differentially expressed proteins were identified and subjected to pathway enrichment and network analyses to characterize obesity-associated alterations. Proteomic profiling showed a significant dysregulation of 456 proteins: 171 upregulated and 285 downregulated. Proteins involved in endoplasmic reticulum quality control particularly endoplasmic reticulum lectin 1 (ERLEC1), were reduced. Conversely, EC-obese demonstrated upregulation of hepatocyte growth factor (HGF), integrin-linked kinase (ILK), CTTNBP2 N-terminal-like protein (CTTNBP2NL), and lysyl oxidase homolog 1 (LOXL1), implicating activation of inflammatory pathways. Bioinformatic analysis showed downregulation of immune-related pathways, including neutrophil degranulation, complement activation in the EC-obese group. ROC analysis identified apolipoprotein(a), phospholipase B-like 1, CTTNBP2NL, and ILK as significant proteins that can differentiate between the obese and non-obese states. Our findings provide insights into obesity-associated proteomic changes in EC and highlight candidate proteins that can be used for molecular stratification after further validation.

## 1. Introduction

Endometrial cancer (EC) is the leading gynecological malignancy worldwide. The age-standardized incidence rate of EC has increased by 0.69% per year globally between 1990 and 2019. Notably, Saudi Arabia has been reported to have one of the highest increases in the incidence rate [1]. There were 360,000 new cases of EC in the year 2021. Projections suggest that the global incidence rate of EC will increase by 6.5% in 2036 when compared to 2021 [2]. While the majority of cases are type 1 EC with a favorable prognosis, a significant subset of cases presents with a high-grade, aggressive form of the disease. The increase in the incidence of EC is thought to be driven by the increasing prevalence of obesity worldwide. Obesity is reported to be associated with 50% of EC cases in North America and Western Europe [3]. The presence of obesity significantly worsens the clinical outcomes in patients with EC. Weight reduction strategies improved the EC prognosis and minimized the surgical complications, highlighting the clinical relevance of obesity-driven mechanisms in EC pathogenesis [4]. Compared with other malignancies, the association of obesity with EC is more significant [5] and has a strong dose–response relationship with the body mass index [6]. This is further substantiated by a recent Mendelian randomization analysis, which found a causal relationship between higher BMI and developing EC [7].

The elevated EC risk in obesity is multipronged and mediated through pathways such as hormonal dysregulation, insulin resistance, and inflammation [8]. The hormonal pathway involves the excessive production of estrogen, facilitated in the adipose tissue by the conversion of the circulating androgens into estrogens [9], leading to a chronic unopposed estrogenic environment. The estrogen conversion rate increases with age and adipose tissue volume and is found to be higher in women with gynoid obesity [10]. Prolonged presence of elevated inflammatory mediators plays a crucial role in the angiogenesis, proliferation, and metastasis of cancers [11]. Elevated adiposity is associated with the upregulation of pro-inflammatory cytokines, such as IL-6 and TNF-α, leading to the activation of NF-κB and STAT3 pathways. STAT3 plays an important role in mediating inflammation-driven carcinogenesis in EC [12]. Obesity-associated insulin resistance results in chronic hyperinsulinemia, which activates the mitogenic signaling via the insulin/IGF axis, reduces the sex hormone-binding globulin, and increases the bioavailable estrogen to drive the endometrial proliferation [8].

Advanced omics studies have provided important insights into the molecular heterogeneity in EC, especially the hormonal and metabolic signaling differences related to obesity. A study found increased PI3K-AKT signaling in obese women with EC in comparison to those without EC and having benign gynecological conditions [13]. In another study, patients with obesity and EC had enhanced estrogen signaling (higher phosphorylated estrogen receptor) and enrichment of gene signatures related to inflammation, immune signaling, and estrogen signaling, whereas non-obese patients exhibited stronger MAPK activity. The study also found that non-obese EC patients had a better prognosis when the PI3K-AKT pathway was activated [14]. A multi-omics analysis categorized patients with EC into metabolism-based subgroups and found that the group with PI3K mutation had a poor prognosis [15]. Despite these advances, important knowledge gaps remain. Previous studies have largely focused on comparing either obese vs. benign conditions [13] or obese vs. non-obese EC patients in a post hoc analysis [14]. An age-matched comparison between obese and non-obese EC patients is essential to describe obesity-specific molecular mechanisms that may underlie differences in tumor biology, prognosis, and therapeutic response. Therefore, the current study was undertaken to comprehensively assess the proteomic profiles of the obese EC patients in comparison to age-matched non-obese EC patients.

## 2. Materials and Methods

### 2.1. Ethical Approval and Consent to Participate

The study protocol was approved by the Institutional Review Board of the College of Medicine, King Saud University (IRB No. E-193622). All procedures were conducted in accordance with institutional guidelines. All participants gave informed consent prior to sample collection. The study was performed at the Proteomics Resource Unit and the Obesity Research Center, College of Medicine, King Saud University, in collaboration with King Khalid University Hospital (KKUH), Riyadh, Saudi Arabia.

### 2.2. Study Design and Patient Selection

Patients attending the outpatient clinics of the Obstetrics and Gynecology-Oncology Department, King Khalid University Hospital, College of Medicine, King Saud University, aged 46–75 years (age-matched), were recruited for the study. To avoid any potential biological effects associated with severe cachexia, we excluded patients with a BMI under 24.9 kg/m^2^. Twenty women diagnosed with endometrial cancer (EC) exhibiting histological atypia with features associated with increased malignant potential were included in the study (*n* = 20). All patients underwent total hysterectomy as part of their clinical management. Approximately 100 mg of endometrial tumor tissue was collected from each patient. Frozen sections were submitted to the pathology department for histopathological confirmation, and fresh tissue samples were snap-frozen in liquid nitrogen and stored at −80 °C until further proteomic analysis. Patient eligibility was assessed during routine clinical visits and participants were enrolled after providing written informed consent. After an overnight fast, 5 mL of venous blood sample was collected from each subject in a plain tube. Serum was separated and stored immediately at −20 °C for further analysis. For all subjects, clinical data were collected including age, systolic blood pressure (SBP), and diastolic blood pressure (DBP). The biochemical assessments including HbA1c and lipid parameters were analyzed using routine laboratory procedures. Demographic data and body mass index (BMI) were calculated as the quotient of weight (kg) divided by height squared (m^2^). From these, BMIs were computed, and women with a BMI of 29.9 kg/m^2^ or higher were categorized as obese, while those between >24.9 and 29.9 kg/m^2^ were considered non-obese [16]. Regarding disease severity, the study population was highly homogeneous, with 80% of participants in both the obese and non-obese groups diagnosed with Stage I endometrial cancer. The patients were divided into two groups (EC-obese *n* = 10, BMI = 38.1 ± 3.87 (range 33.7–47.0) and EC-Non-obese (*n* = 10, BMI = 28.1 ± 1.24 (range 25.8–29.8) with *p*-Value = <0.0001).

### 2.3. Sample Preparation for Proteomics

#### Protein Extraction and Digestion

Proteins were extracted from 100 mg tissue samples. The tissue samples were first suspended in 1× PBS to remove blood clots. Tissue samples were homogenized directly in 0.5 mL of lysis buffer (pH 8.8, containing 30 mM Tris-HCl, 7 M urea, 2 M thiourea, 4% CHAPS, and 1× protease inhibitor mix) on ice using a T25 digital ULTRA TURRAX homogenizer (IKA, Staufen im Breisgau, Germany). After homogenization, samples were vortexed for 2 min, then shaken for 1 h at 450 rpm at 25 °C before sonication (Qsonica Microsonicator, Newtown, CT, USA; 30% pulse, two 1-min intervals separated by a 1 min gap). Protein extracts were clarified by centrifugation (16,000× *g*, 40 min, 4 °C) followed by precipitation using ice-cold acetone with overnight incubation at −20 °C to precipitate proteins. Samples were centrifuged at 14,000× *g* for 15 min, and the supernatant was discarded. The protein pellet was resuspended in 2× lysis buffer. Protein concentration was measured in triplicate using the 2D-Quant Kit according to the manufacturer’s instructions (GE Healthcare, Chicago, IL, USA) [17,18,19].

Protein samples (50 μg) were denaturated in 10 μL of 6 M urea, reduced with 10 μL dithiothreitol 200 mM (30 min at 60 °C) and alkylated by adding 1 µL of iodoacetamide 400 mM (30 min in the dark). After dilution with ammonium bicarbonate buffer 50 mM (65 uL) were digested overnight at 37 °C with 2.5 µL of sequencing-grade modified Trypsin (1 µg/μL; Promega Corporation, Madison, WI, USA). The reaction was acidified with 7 µL of 10% formic acid and the samples were desalted using Pier-e C18 spin columns (Thermo Scientific, Mount Prospect, IL, USA). Following quantification with a Pierce Quantitative Colorimetric Peptide Assay (Thermo Scientific, Mount Prospect, IL, USA), the eluted peptides were dried using vacuum centrifugation (Eppendorf Concentrator plus TM, Hamburg, Germany) [20,21,22].

### 2.4. Liquid Chromatography and Mass Spectrometry

In the proteomics workflow, peptides were reconstituted in 0.1% (*v*/*v*) formic acid and processed through a Dionex UltiMate 3000 nano-LC system (Thermo Fisher Scientific, Germering, Germany) with a WPS-3000 autosampler (Thermo Fisher Scientific, Germering, Germany). The samples are concentrated on a PepMap100 C18 trap column (Thermo Fisher Scientific, Germering, Germany) before undergoing high-resolution separations on a 50 cm C18 analytical column. Following separation, the peptides undergo ionization via a nanospray source and are analyzed by a Q Exactive Plus Hybrid Quadrupole-Orbitrap mass spectrometer (Thermo Fisher Scientific, Waltham, MA, USA). The system operates in positive ion mode using nano electrospray (nESI) potential at 2000 V, maintained within a maximum duty cycle of 3 s. The MS spectra were acquired across a range of 375–1650 *m*/*z* at a resolution of 70,000, while MS/MS fragmentation scans were performed at a resolution of 17,500 with a fixed starting mass of 80 *m*/*z*. Mass spectra were acquired in a data-dependent acquisition (DDA) mode, using a 20 s dynamic exclusion window to maximize proteome coverage. To maintain optimal signal intensity, the automatic gain control was set to 3 × 10^6^ and 1 × 10^5^ for MS and MS/MS scans, respectively [20,21,22].

### 2.5. Data Analysis

Raw MS and MS/MS data were analyzed using Proteome Discoverer version 3.0 (Thermo Fisher Scientific, Bremen, Germany) with the Sequest HT (as integrated in Proteome Discoverer 3.0) search engine. Peptide identification was performed against the HUMAN-refprot-isoforms.fasta database. Search settings were as follows: precursor and fragment mass tolerances were set to 15 ppm and 0.02 Da, respectively; a maximum of two missed cleavages was allowed; trypsin (full) was specified as the digestion enzyme; dynamic modifications included N-terminal acetylation and methionine oxidation; and carbamidomethylation of cysteine was set as a fixed modification. Peptide-spectrum matches (PSMs) were filtered using a false discovery rate (FDR) of 1% at both the peptide and protein levels. A minimum of two unique peptides per protein was required for identification. The filtered PSM data were exported and organized in Microsoft Excel. Multivariate statistical analysis was performed using MetaboAnalyst version 6.0 (McGill University, Montreal, QC, Canada; http://www.metaboanalyst.ca, accessed on 24 June 2025). Only proteins identified and quantified using label-free quantification (LFQ) intensity values were included in subsequent analyses.

Data processing included median normalization, Pareto scaling, and log transformation [23]. Principal Component Analysis (PCA) was performed for visualization of study groups and outlier detection, and an Orthogonal Partial Least Squares Discriminant Analysis (OPLS-DA) model was also generated for supervised and unsupervised models. To ensure the statistical validity and predictive reliability of the supervised multivariate analysis, the OPLS-DA model was further evaluated using a 5-fold internal cross-validation to determine goodness of fit (R2Y) and predictive ability (Q2), and a 100-cycle permutation test was conducted to confirm that the observed separation was not due to random chance [20,21,22].

### 2.6. Statistical Analysis and Functional Annotations

Demographic and clinical parameters are presented as a mean ± standard deviation. Comparisons of two groups were performed using the Student’s *t*-test. A *p* < 0.05 was considered statistically significant. All statistical analyses were performed using SPSS (v.26.0; IBM Corp., Armonk, NY, USA). Statistical comparisons between groups were conducted using Student’s *t*-test within the Proteome Discoverer v3.0 framework. Significance was defined by an FDR-adjusted *p*-value (q-values) below 0.01. A post hoc power analysis was conducted using a two-sample *t*-test framework on log_2_-transformed LFQ intensities. Assuming a protein-level standard deviation of 0.4 and α = 0.05, a sample size of *n* = 10 per group provides approximately 80% statistical power to detect expression differences ≥ 1.5-fold (see Supplementary Note S1). Proteins with smaller fold changes may be underpowered and should be interpreted as preliminary observations. Differentially expressed significant proteins were filtered using a threshold of an FDR-adjusted *p*-value ≤ 0.05 and a fold change ≥ 1.5. Functional enrichment and pathway modeling were performed using Ingenuity Pathway Analysis (IPA) (QIAGEN Inc., Hilden, Germany; analysis portal) with quantitative datasets imported for core analysis that determines functions and pathways associated with the protein list. Furthermore, the PANTHER (protein analysis through evolutionary relationships) classification system (http://www.pantherdb.org, accessed on 15 July 2025) was used for categorizing the identified proteins by their molecular function and biological process [20,21,22].

## 3. Results

The clinical and metabolic characteristics of the study participants are summarized in Table 1. Comparisons between the EC-Non-obese and EC-obese groups revealed that both cohorts were well-matched for age, blood pressure, and lipid profiles, with no statistically significant differences observed. Notably, the HbA1c levels in both groups fell within the pre-diabetic to diabetic ranges. Despite these elevated glycemic markers, all participants were managing their blood glucose levels through dietary modifications alone and were not on any glucose-lowering medications at the time of the study. As expected by the study design, BMI was significantly higher in the EC-obese group (38.1 ± 3.87 kg/m^2^) compared to the EC-Non-obese group (28.12 ± 1.24 kg/m^2^); *p* < 0.001.

### 3.1. Label-Free Quantitative

To compare obese and non-obese EC patient cohorts, we used a label-free quantitative proteomics strategy. This analysis yielded a total of 6331 non-redundant proteins, with each quantification supported by two or more unique peptides to ensure high-confidence identification. Significant and differentially expressed proteins (fold change greater than 1.5 or less than 0.66, *p*-value < 0.05) between the EC-Non obese and EC-obese groups were identified as 456 proteins (171 and 285 up- and downregulated) (Table S1).

### 3.2. Analysis of Differentially Expressed Proteins in Obese and Non-Obese Patients with EC

**Figure 1:**
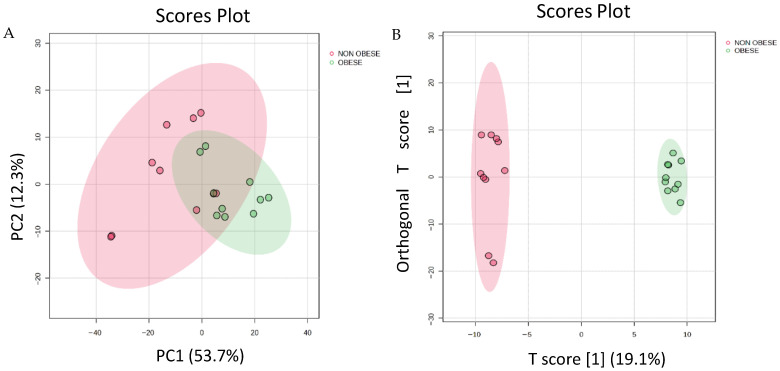
(**A**) Principal component analysis (PCA) plot of the two principal components. (**B**) Orthogonal partial least squares discriminant analysis (OPLS-DA) shows a clear separation between the two groups, indicating a significant proteomic difference between obese and non-obese patients with EC. The robustness of the created models was evaluated by the fitness of the model (R2Y = 0.99) and predictive ability (Q2 = 0.785) values in a larger dataset (*n* = 100).

Proteins that differentiated the two study groups are presented in Figure 1. Principal component analysis (PCA) was performed to visualize group separation and to assess potential outliers. The score plot shown in Figure 1 illustrates the distribution of the two groups based on their proteomic profiles. The PCA score plot showed clear separation between obese and non-obese cohorts with endometrial cancer, indicating that the proteomics profiles of these two groups significantly differed. An orthogonal partial least squares discriminant analysis (OPLS-DA), a supervised multivariate approach, was applied and displayed in an OPLS-DA model score plot to maximize group separation based on proteins meeting our selection criteria (*p*-value ≤ 0.05 and FC ≥ 1.5) (Figure 1B). An unsupervised PCA followed by partial least squares discriminant analysis (PLS-DA) and Orthogonal PLS-DA (OPLS-DA), confirming model stability through rigorous cross-validation and permutation testing, provides a high degree of confidence in the statistical validity and reliability of the identified proteins. The model demonstrated a clear, evident, and significant separation between the two groups (R2 = 0.99 and Q2 = 0.785), indicating variations in proteomic expression between the obese and non-obese groups, as shown in Figure S1.

### 3.3. Protein Dysregulation in Response to Altered Body Mass Index in Endometrial Cancer

**Figure 2:**
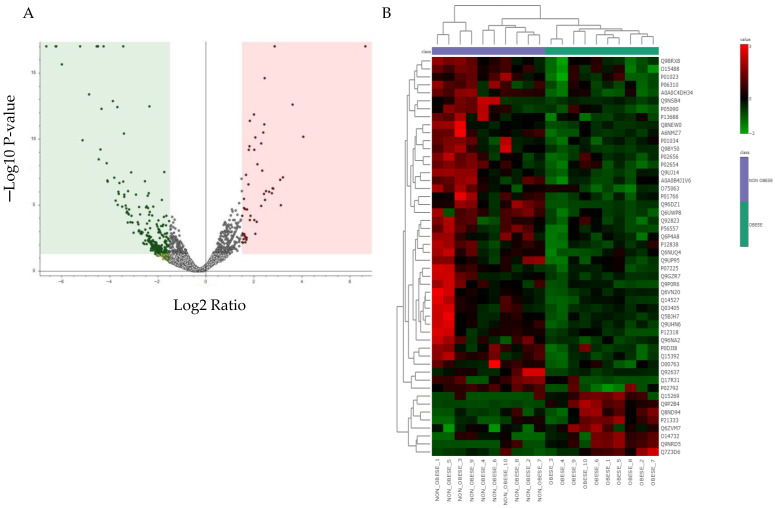
(**A**) The volcano plot showed a significant change in the levels of several proteins in obese and non-obese patients with endometrial cancer. The green dots represent downregulated and red dots represent upregulated proteins in groups. The grey dots represent statistically non-significant proteins (unpaired *t*-test, FDR *p*-value ≤ 0.05, fold change ≥ 1.5). (**B**) Hierarchical Clustering (HAC) and heat map analysis of identified proteins that significantly altered between obese and non-obese patients with endometrial cancer samples. The color range bar indicates downregulated proteins as green and upregulated proteins as red.

Volcano plot analyses were conducted to identify significantly dysregulated proteins in obese and non-obese patients with endometrial cancer. Proteins were considered significantly differentially expressed if they exhibited a fold change greater than 1.5 or less than 0.66 with an associated *p*-value < 0.05. As depicted in Figure 2A, differences in BMI in endometrial cancer resulted in the significant dysregulation of 456 proteins. Of these, 171 proteins were significantly increased (red), whereas 285 proteins were significantly decreased (green) in obese compared with non-obese endometrial cancer tissues. The heat map in Figure 2B displays the top 100 proteins with the most pronounced differences between the two groups. These proteins were subsequently analyzed to investigate molecular, biological, and cellular alterations associated with variation in body mass index.

### 3.4. Evaluation of Proteins Between Obese and Non-Obese Groups with Endometrial Cancer

**Figure 3:**
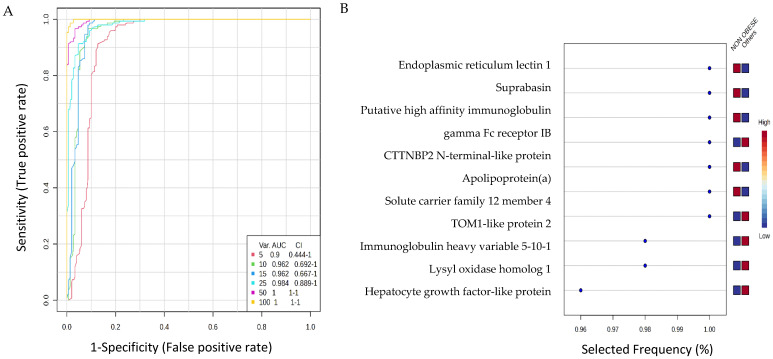
Biomarker evaluation on obese and non-obese groups with endometrial cancer. (**A**) Biomarker evaluation on obese and non-obese groups with endometrial cancer. (**A**) Receiver Operating Characteristic (ROC) curves derived from the PLS-DA model. The plot illustrates the diagnostic performance in distinguishing obese and non-obese using panels of increasing size. The Area Under the Curve (AUC) and 95% Confidence Intervals (CI) are provided for models incorporating the top 5, 10, 15, 25, 50, and 100 protein features, demonstrating the relationship between feature number and predictive accuracy. (**B**) Frequency plot showing the top 10 significantly dysregulated identified proteins in the obese and non-obese groups.

The potential of the identified proteins to serve as biomarkers was assessed using multivariate exploratory ROC analysis, particularly partial least squares discriminant analysis (PLS-DA) as a method for both classification and feature ranking. Six features in the ROC curve obtained from PLS-DA and cross-validation (CV) had an Area Under the Curve (AUC). The ROC curve showed a set of variant proteins (5, 10, 15, 25, 50, and 100), with different AUCs and confidence intervals (CIs) (Figure 3A). Among the top 10 significantly dysregulated proteins, CTTNBP2 N-terminal-like protein, TOM1-like protein 2, sImmunoglobulin heavy variable 5-10-1, Lysyl oxidase homolog 1, Hepatocyte growth factor-like protein were up-regulated in the obese group, whereas Endoplasmic reticulum lectin 1, Suprabasin, Putative high-affinity immunoglobulin gamma Fc receptor IB, Apolipoprotein(a), Solute carrier family 12 member 4 were downregulated in the obese group in comparison to the non-obese group (Figure 3B).

### 3.5. Evaluation of the Top Proteins Identified Between the Obese and Non-Obese Groups with Endometrial Cancer

**Figure 4:**
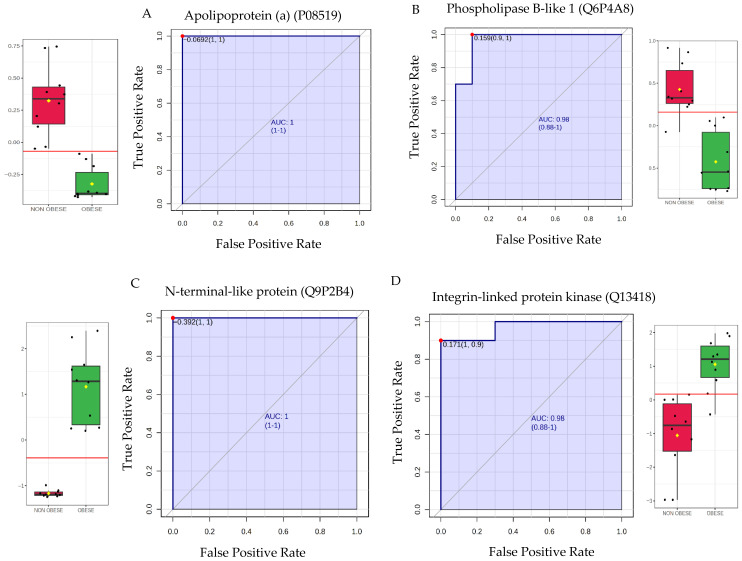
Dysregulated proteins in endometrial cancer patients by obesity status. The top dysregulated proteins with the highest Area Under the Curve (AUC) values for distinguishing between obese and non-obese endometrial cancer groups. The horizontal red line within each box plot indicates the optimal diagnostic cutoff point. (Sensitivity, plotted on the *y*-axis as true positive rate) and (1-Specificity, plotted on the *x*-axis as false positive rate). In Box and whisker red box represents the non-obese group, while green represents the obese group. The red line within each box plot indicates the optimal cutoff point for the biomarker, maximizing both sensitivity and specificity. (**A**) Apolipoprotein(a) (P08519): Top downregulated protein in the obese group (AUC = 1.00). Box plot shows significant difference (FDR *p* ≤ 0.05) and a fold change ≥ 1.5. (**B**) Phospholipase B-like 1 (Q6P4A8): Second top downregulated protein in the obese group (AUC = 0.98). Box plot shows significant difference (FDR *p* ≤ 0.05) and a fold change ≥ 1.5. (**C**) CTTNBP2 N-terminal-like protein (Q9P2B4): Top upregulated protein in the obese group (AUC = 1.00). Box plot shows significant difference (FDR *p* ≤ 0.05) and a fold change ≥ 1.5. (**D**) Integrin-linked protein kinase (Q13418): Second top upregulated protein in the obese group (AUC = 0.98). Box plot shows significant difference (FDR *p* ≤ 0.05) and a fold change ≥ 1.5.

The identified proteins were evaluated via ROC curves, and for Apolipoprotein(a) (P08519) (Figure 4A) and Phospholipase B-like 1 (Q6P4A8) (Figure 4B), both showed an AUC of 1.00 and 0.98 respectively, and both were found to be downregulated in the obese group. Conversely, CTTNBP2 N-terminal-like protein (Q9P2B4) (Figure 4C) and Integrin-linked protein kinase (Q13418) (Figure 4D) had AUC values of 1.00 and 0.98, respectively, and were upregulated in the obese group.

**Figure 5:**
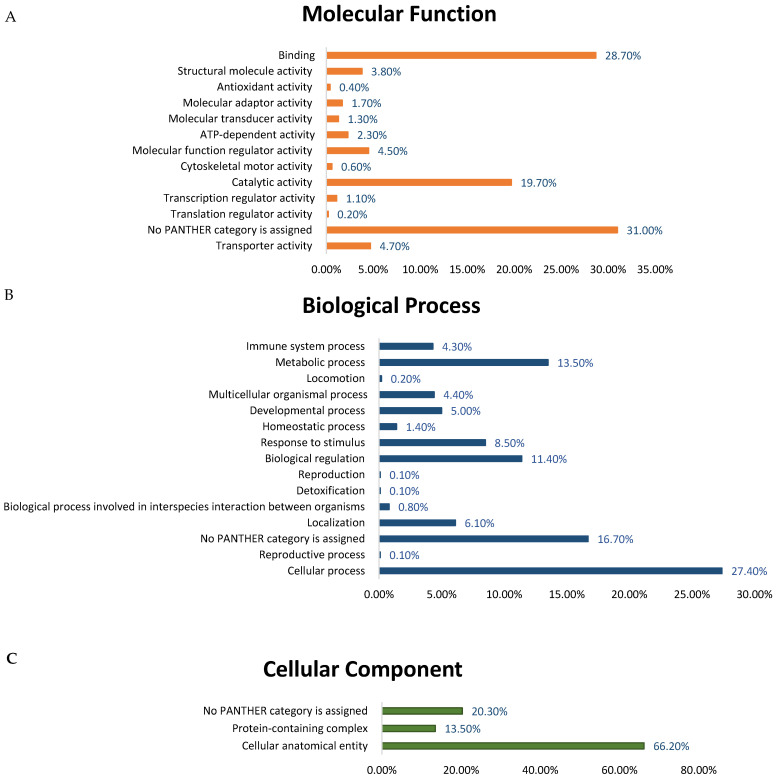
The identified proteins were classified according to Gene Ontology terms, including (**A**) molecular function, (**B**) biological process, and (**C**) cellular components.

Proteins were classified using protein analysis through evolutionary relationships (PANTHER) classification system by their molecular functions (Figure 5A), biological processes (Figure 5B), and cellular components (Figure 5C). Most of the differentially expressed proteins identified by molecular function were enzymes with binding (28.7%), followed by catalytic activity (19.7%) (Figure 5A). With regard to biological processes, the identified proteins were involved in cellular processes (27.4%), metabolic processes (13.5%) and biological regulation (11.4%) (Figure 5B). In terms of cellular localization, the majority of the identified proteins were associated with the cellular anatomical entity (66.2%), followed by the protein-containing complex (13.5%) (Figure 5C).

### 3.6. Interaction Network Analysis of Differentially Expressed Proteins

**Figure 6:**
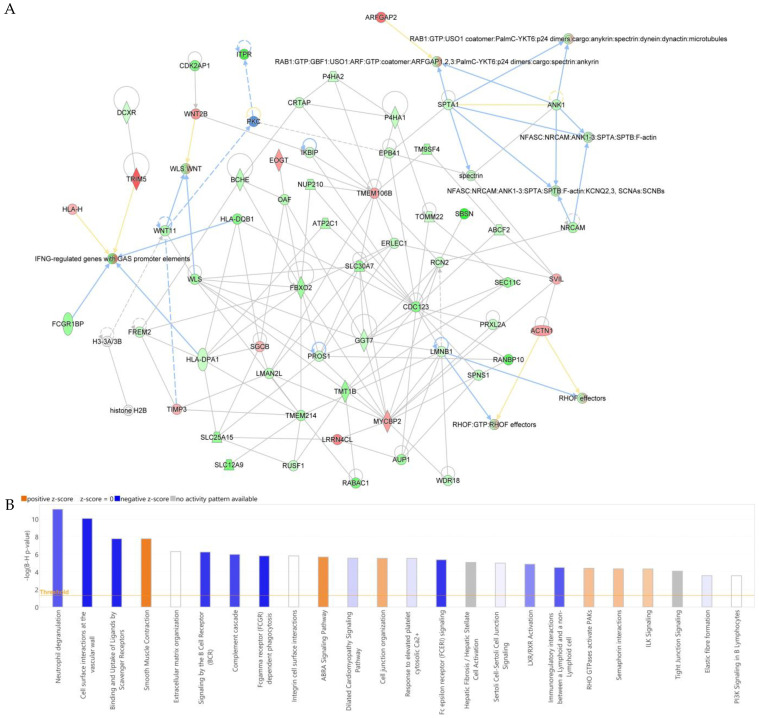
(**A**) Schematic of top network pathways from differentially regulated proteins in obese and non-obese patients with endometrial cancer. Nodes colored blue represent downregulation and orange represents upregulation. (**B**) IPA (QIAGEN Inc., Hilden, Germany) identified and ranked the most significant canonical pathways by *p*-value, from which interaction networks were generated. Darker intensity of orange color indicates strong prediction of pathway activation and darker intensity of blue color represent strong prediction of pathway inhibition.

Ingenuity Pathway Analysis (IPA) was used to interpret the biological relevance of the differentially expressed proteins. IPA generates interaction networks by assessing the overlap between the input protein dataset and curated biological knowledge databases. The resulting protein–protein interaction network is shown in Figure 6. IPA identified cancer, cellular development, cellular growth, and proliferation as the highest-scoring functional network distinguishing obese and non-obese endometrial cancer tissues (score = 103) (Figure 6A; Table S2; Supplementary Data S1). The top canonical pathways included cell surface interactions at the vascular wall, ligand binding and uptake by scavenger receptors, and Fc gamma receptor–mediated phagocytosis, which showed negative z-scores. In contrast, smooth muscle contraction, the ABRA signaling pathway, and cell junction organization were associated with positive z-scores (Figure 6B).

## 4. Discussion

In this study, we compared tissue-based proteomic profiles between obese and non-obese patients with EC using label-free quantitative proteomics analysis. A total of 6331 non-redundant proteins were quantified, of which 456 were significantly differentially expressed between groups. Among these, 171 proteins were upregulated, and 285 proteins were downregulated. The PCA plot indicates a clear separation between obese and non-obese patients with EC samples. This suggests that the most significant variance in the dataset is driven by the differences in protein expression related to obesity status. Functional analysis based on Gene Ontology demonstrated that the differentially abundant proteins were mostly involved in cellular processes, metabolic processes, and biological regulation functional categories [24]. Furthermore, the Receiver Operating Characteristic (ROC) curve generated by PLS-DA found several significantly dysregulated proteins with strong biomarker potential.

Pathway enrichment analysis revealed that neutrophil deregulation was the most significantly downregulated pathway in obese EC tissues. Neutrophils are key regulators of innate immune activation in obesity-associated inflammation by infiltrating adipose tissue during early inflammatory stages, which in turn promotes low-grade inflammation and reduces insulin signaling [25,26]. Neutrophils also play a role in the tumor microenvironment via remodeling the extracellular matrix, creating conditions that assist tumor invasion and metastasis [27,28,29]. The suppression of the key immune pathways, such as B cell receptor signaling, complement activation, and Fc receptor-mediated phagocytosis, suggests a pro-inflammatory and tumorigenic environment [29]. Clinically, this altered immunological landscape with diminished innate and humoral immune presence suggests that obese patients with EC may exhibit a highly immunosuppressed tumor microenvironment.

At the protein level, several key molecules were downregulated in obese EC tissues, reflecting obesity-associated changes in tumor biology. Suprabasin (SBSN) is a known oncoprotein and a potential biomarker in various cancers, including salivary adenoid cystic carcinoma, lung carcinoma, and myelodysplastic syndromes [30]. Its overexpression has been linked to increased cell proliferation, migration, angiogenesis, and resistance to apoptosis [30]. However, SBSN was shown to be downregulated in EC tissues. In tissue samples, both isoforms 1 and 2 were downregulated, whereas in serum, isoform 1 was upregulated and isoform 2 remained downregulated. SBSN was found to be a potential biomarker for EC [31]. The Cancer Genome Atlas program data also showed a downregulation of SBSN in EC tissues [32]. Our results showed that suprabasin was downregulated in obese endometrial cancer tissue samples. As our comparison is obese vs. non-obese among patients with EC, the downregulation of SBSN protein in patients with obesity and EC indicates that the presence of obesity led to further downregulation of the SBSN protein. It seems that the tumor pathology is augmented by the obesity-related physiological changes. Further studies are necessary to investigate the precise molecular mechanisms behind the downregulation of SBSN protein in the presence of obesity in EC.

Apolipoprotein(a) [Apo(a)], which forms lipoprotein(a) through covalent binding to apolipoprotein B-100, was also significantly downregulated in obese EC tissues. Apo(a) is classically associated with cardiovascular disease; emerging findings have linked it to cancer and obesity-related carcinomas. Apo(a)’s role in cancer depends on the type of cancer. Decreased Lp(a) levels are associated with shorter recurrence-free and overall survival in patients with hepatocellular carcinoma (HCC) [33]. Similarly, a study on a Japanese cohort found that reduced Lp(a) concentrations were linked with all-cause and cancer mortality [34]. Estrogen levels are long known to suppress the expression of apo(a) levels. Patients with obesity are reported to have high adipose tissue-mediated estrogen production [9]. The high estrogenic environment likely led to the lower expression of apo(a) in EC tissues. Apo(a) is also reported to have an anti-tumor effect in vitro. Therefore, the obesity mediated lower expression of apo(a) might have a role to play in the obesity-associated changes in the tumor biology.

Endoplasmic Reticulum Lectin 1 (ERLEC1), a key component of the ER-associated degradation pathway, plays a fundamental role in protein quality control and is also significantly downregulated in obese EC patients. Cancer cells typically survive in hostile environments, which are usually characterized by nutrient deprivation, inflammation, and hypoxia. Evidence suggests that reduced expression of ER quality control proteins is associated with the adaptability and survival of cancer cells in such hostile environments [35]. Downregulation of ERLEC1 could lead to the accumulation of misfolded proteins, causing the unfolded protein response (UPR) to be activated. Low-nutrient conditions or nutrient oversupply, as in the case of obesity, also induce the ER stress and chronic UPR activation. UPR induction facilitates the cancer cell adaptability by activating autophagy and modulating inflammatory responses [35]. In addition, cancer cells also selectively suppress ER quality control proteins to avoid apoptosis signaling and maintain specific UPR pathways, such as PERK and IRE1α, which promote tumor growth and stress tolerance [36,37]. Consistent with this, Zhang et al. demonstrated that ER stress-associated genes are dysregulated in endometrial carcinoma and promote progression of the disease [38].

Obesity exacerbates suppression of ER quality control proteins by imposing a chronic overload in the ER. The ER stress activates PERK and IRE1α pathways to selectively suppress ER quality control proteins, which would otherwise lead to the induction of apoptosis [37]. Chronic obesity leads to selection of tolerance of misfolded proteins and not correction. Experiments show that under conditions like obesity and insulin resistance, the ER becomes overloaded with unfolded proteins due to the functional exhaustion of ER quality control proteins. Downregulation of ERLEC1, an ER quality control protein, can lead to the accumulation of misfolded proteins, thereby amplifying insulin resistance, inflammatory cytokine release, lipogenesis, and hepatic steatosis [39,40]. Although direct evidence that links ERLEC1 downregulation to obesity is limited, chronic ER stress and UPR activation are associated features of obesity and insulin resistance. Collectively, these observations indicate that ERLEC1 downregulation is likely to be an obesity-driven proteostasis-related adaptation in EC, linking the metabolic stress to tumor survival mechanisms.

Phospholipase B-like 1, a transcription factor also known as Phospholipase B Domain-Containing Protein 1 (PLBD1), was also downregulated in obese tissues. PLBD1 exhibits weak phospholipase activity, and its role in obesity and cancers is not clear. It is reported to mediate the proliferation and invasive ability of glioma cells [41]. However, in patients with EC, lower expression of PLBD1 is associated with a poor patient survival probability [42]. PLBD1 levels are significantly lower (*p* = 0.001) in our study participants who had obesity and EC when compared with those who had only EC. Our results also show that PLBD1 can be a potential biomarker for EC in patients with obesity. As downregulation of PLBD1 is known to be associated with poor survivability in EC patients, the presence of obesity would likely lead to a poor prognosis. Further long-term studies are necessary to elucidate the role of the PLBD1 in obese patients with EC.

In contrast to the downregulated proteins, several proteins were significantly upregulated in obese tissues, demonstrating activation of tumorigenic and stress-adaptive pathways. Integrin-linked kinase (ILK), a central mediator of integrin signaling, was among the most prominently upregulated proteins. ILK mediates the interactions between the cell and the extracellular matrix and plays a key role in promoting cell growth, survival, migration, and invasion in cancer cells [43]. ILK is expressed in normal endometrium to enhance the receptivity of the endometrium, which is a prerequisite for the implantation of the embryo. However, its overexpression promotes proliferation and invasion of EC cells [44]. It is known to be a linking molecule between the integrins and the actin cytoskeleton. Integrin-ILK signaling is essential for the formation of invadopodium, an actin-rich protrusion that enables cell movement and metastasis in invasive tumor cells [29]. ILK is also involved in insulin signaling. Activation of Akt, a crucial molecule in the insulin signaling cascade, requires ILK. A study found that ILK is critical for the insulin resistance mediated by a high-fat diet in mice [45]. However, there are no studies showing that the ILK expressed in the endometrium plays a role in obesity or the insulin pathway. As our study compared the protein levels between EC patients with and without obesity, the overexpression of ILK in EC patients with obesity is likely to have been caused by the underlying obesity. Our results demonstrate that ILK emerges as one of the key proteins distinguishing obese from non-obese patients with EC.

Hepatocyte growth factor (HGF), also known as scatter factor (SF), is another significantly upregulated protein. It is a mesenchyme-derived growth factor that exerts its biological effects by activating the receptor tyrosine kinase c-Met (MET), leading to enhanced cell proliferation, motility, morphogenesis, and survival. And high expression of HGF and its receptor c-Met in tumor cells, together with increased HGF production by stromal fibroblasts, is associated with higher tumor grade, deeper myometrial invasion, and greater metastatic potential in endometrial carcinoma [46,47,48]. HGF is also closely linked to obesity and metabolic dysregulation. Circulating and tissue levels of HGF are elevated in obese individuals and correlate positively with body mass index, insulin resistance, and features of the metabolic syndrome [48]. Adipose tissue has been identified as a significant source of HGF, suggesting that obesity may create a systemic and local pro-tumorigenic microenvironment through sustained activation of HGF/c-Met signaling. Preclinical studies showed that HGF signaling may exert a protective effect and support the pancreatic β-cells survive and function under metabolic stress, suggesting a context-dependent dual role for HGF [49]. In obesity-associated EC, chronic activation of the HGF/c-Met pathway may preferentially promote tumor cell survival, invasiveness, and anoikis resistance. These data are consistent with our finding that HGF is upregulated in obese EC tissue compared with non-obese EC samples.

CTTNBP2 N-terminal-like protein (CTTNBP2NL), a component associated with the STRIPAK (Striatin-interacting phosphatase and kinase) complex, was also upregulated in EC tissues [50]. STRIPAK complexes function as signaling scaffolds that incorporate multiple kinases and phosphatases, regulating several key signaling pathways involved in cell proliferation, apoptosis, and tissue homeostasis, such as Hippo and MAPK [50]. Dysregulation of STRIPAK-mediated signaling has been implicated in oncogenic processes such as tumor cell migration, invasion, and growth [50]. Although the precise functional role of CTTNBP2NL within the STRIPAK complex remains unclear, its association with STRIPAK components suggests that it may influence the STRIPAK-dependent signaling outputs. As alterations in the expression of individual STRIPAK-associated proteins modulate pathway activity and cellular behavior [51], CTTNBP2NL upregulation could indirectly affect tumor-relevant signaling networks. However, there is currently no direct evidence linking CTTNBP2NL expression to obesity or metabolic disease. The STRIPAK complex has been reported to control pathways like Hippo and MAPK, which are sensitive to metabolic stress and inflammation. Therefore, enhanced CTTNBP2NL level might be seen as a protective or pathological response to the metabolic consequences of obesity. While the consequences of CTTNBP2NL upregulation in EC remain unclear, our findings suggest that obesity may influence tumor biology through modulation of signaling scaffolds such as STRIPAK. Further mechanistic studies will be required to elucidate the specific role of CTTNBP2NL in STRIPAK function and to clarify its contribution to obesity-associated endometrial carcinogenesis.

Lysyl oxidase homolog 1 (LOXL1), a copper-dependent amine oxidase involved in the cross-linking of the extracellular matrix (ECM), was also significantly upregulated in obese EC tissues. It has been implicated in cancer progression through its effect on cancer cells as well as on the cancer microenvironment. LOXL1 activity can activate integrin/FAK/PI3K/AKT pathways and may interact with TGF-β signaling, thereby amplifying tumor progression [52]. LOXL1-mediated remodeling of collagen and elastin alters ECM stiffness, which in turn activates integrin–FAK signaling, leading to downstream activation of PI3K/AKT and MAPK cascades that support tumor cell survival, proliferation, and invasion [52,53]. The role of LOXL1 in cancers is largely context-dependent. In the early stages, loss of LOXL1 disrupts the ECM integrity and tissue homeostasis, potentiating early tumor initiation, whereas in established tumors, its overexpression promotes tumor invasion, epithelial–mesenchymal transition, and immune evasion [54]. For example, in colorectal cancer, LOXL1 is upregulated and associated with increased tumor aggressiveness, activation of integrin/FAK/MAPK signaling, and suppression of CD8^+^ T-cell infiltration [52]. In addition, LOXL1 levels can increase when integrins interact with collagen, and its expression is further controlled by TGF-β signaling in the stroma and cancer-associated fibroblasts [53].

Overall, these proteomic comparisons between obese and non-obese patients with endometrial cancer reveal that obesity is associated with distinct and coordinated alterations in immune regulation, metabolic stress responses, extracellular matrix remodeling, and oncogenic signaling pathways within the tumor microenvironment. At the protein level, obesity was associated with downregulation of putative tumor-suppressive or context-dependent proteins such as suprabasin, apolipoprotein(a), PLBD1, and the ER quality control protein ERLEC1, linking metabolic stress and impaired proteostasis to enhanced tumor adaptability. The dysregulated proteins identified in our study particularly Apo(a), PLBD1, ILK, HGF, CTTNBP2NL, and LOXL1 offer significant translational potential for obesity-associated EC [55,56,57,58,59,60]. The upregulation of HGF, ILK, CTTNBP2NL, and LOXL1 highlights activation of PI3K/AKT, MAPK, Hippo, and ER stress-associated survival pathways that promote invasion, anoikis resistance, and metastatic potential. The high AUC values observed in our ROC analysis suggest that these markers are robust candidates for clinical implementation. Specifically, Apo(a) and HGF are measurable in circulation and thus represent feasible candidates for minimally invasive circulation-based assays [61,62]. In contrast, ILK, PLBD1, CTTNBP2NL, and LOXL1 may be more suitable for assessment via immunohistochemistry or even by targeted proteomic panels using biopsy or surgical tissue samples. In comparison to conventional markers such as CA125 and HE4 for EC, which often show limited specificity in the context of high adiposity, the proteins identified here provide distinct prognostic and predictive value by reflecting the metabolic and extracellular matrix remodeling inherent to the obese tumour microenvironment [63].

It is important to note that several dysregulated proteins identified here can also represent therapeutic targets. For instance, inhibitors of the HGF/c-Met axis and ILK-mediated PI3K/AKT signaling are currently under clinical investigation and could form the basis for pathway-directed therapies [64,65]. Collectively, these findings support that obesity reshapes the endometrial cancer proteome toward a stress-tolerant phenotype, and provide mechanistic insight into the adverse prognostic impact of obesity in EC. Furthermore, by integrating these obesity-related proteins into personalized treatment, clinicians may be able to stratify high-risk individuals and select targeted interventions.

The major strengths of this study include the use of tissue-based, label-free quantitative proteomics to comprehensively characterize obesity-associated molecular differences in endometrial cancer. The clear separation of obese and non-obese EC samples by multivariate analysis further underscores the robustness of the observed proteomic differences. However, several limitations should be acknowledged. The relatively modest sample size may limit the detection of low-abundance proteins and proteins with modest expression changes and preclude detailed stratification by histological subtype or TCGA molecular class. Because patients with a BMI < 24.9 kg/m^2^ were excluded to avoid cancer-related cachexia cases, the nonobese group comprised individuals in the overweight range. Consequently, this may have attenuated the observed magnitude of obesity-associated alterations in the EC patients. The cross-sectional design prevents causal inference between obesity and protein expression changes, and functional validation of key findings was beyond the scope of this study. Therefore, these results warrant cautious interpretation. We cannot currently ascertain whether the observed proteomic shifts are a precursor to tumor development, a concurrent manifestation, or a consequence of the tumor microenvironment. Future longitudinal investigations are necessary to elucidate the precise temporal relationship between obesity associated protein dysregulation and EC progression. Another major limitation is the lack of independent validation for significantly identified proteins. This is an exploratory discovery phase study. Our findings are preliminary and require independent validation using orthogonal methods such as western blotting or immunohistochemistry. Future studies with larger cohorts will be essential to validate these findings and to clarify the mechanistic and clinical relevance of obesity-driven proteomic remodeling in endometrial cancer.

The findings from our present study can contribute clinically to improve molecular stratification of EC by identifying a subgroup characterized by obesity-driven immune modulation and metabolic pathway activation. The proteins identified in this study were derived directly from EC tissue and reflect obesity-associated molecular alterations within the tumor microenvironment. Although tissue-based discovery does not automatically translate to circulating levels, it is known that certain proteins such as apolipoprotein(a) and HGF are detectable in blood and may be explored in future validation studies as minimally invasive indicators. The strong discriminatory performance observed in ROC analysis suggests potential value in distinguishing obesity-associated EC, while associations of PLBD1, HGF, and LOXL1 with aggressive tumor features indicate possible prognostic relevance [52]. Furthermore, several dysregulated proteins including LOXL1 are linked to therapeutically targetable signaling networks that can lead to potential drug development efforts. LOXL1 inhibitors could disrupt the tumor’s ability to metastasize and evade the immune system, supporting their potential relevance for future precision-based management strategies in obesity-associated EC, pending further validation [37].

## 5. Conclusions

Our findings demonstrate that obesity is associated with distinct proteomic remodeling in EC. The significantly altered proteins provide insights into the mechanisms that are linked to key oncogenic and stress-adaptation pathways, such as PI3K/AKT, MAPK, Hippo, and unfolded protein response signaling. The identified proteins can be used for molecular stratification and risk determination.

## Figures and Tables

**Table 1:** Comparison of demographic and clinical parameters between EC obese and EC-Non-obese patients.

	EC-Non-Obese Mean ± SD *n* = 10	EC-Obese Mean ± SD *n* = 10	*p* Value
Age (Years)	62.85 ± 6.49	59.56 ± 13.57	0.565
SBP (mmHg)	127.42 ± 7.63	137.25 ± 16.20	0.167
DBP (mmHg)	65.14 ± 6.79	69.75 ± 7.45	0.236
BMI (kg/m^2^)	28.12 ± 1.24	38.1 ± 3.87	<0.001 *
HbA1c %	6.6 ± 1.40	6.9 ± 1.56	0.701
Triglycerides (mmol/L)	1.07 ± 0.49	1.60 ± 0.94	0.209
LDL Cholesterol (mmol/L)	2.49 ± 1.10	2.29 ± 0.95	0.761
HDL Cholesterol (mmol/L)	1.34 ± 0.44	1.18 ± 0.30	0.530
Total Cholesterol (mmol/L)	4.32 ± 1.38	4.24 ± 0.924	0.915

SBP, systolic blood pressure; DBP, diastolic blood pressure; BMI, body mass index; HbA1c, hemoglobin A1c; LDL, low-density lipoprotein; HDL, high-density lipoprotein. Data are presented as mean ± standard deviation and compared by Student’s *t*-test. Values of * *p* < 0.05 were considered significant.

## Data Availability

The original contributions presented in this study are included in the article/Supplementary Materials. Further inquiries can be directed to the corresponding author.

## Supplementary Materials

The following supporting information can be downloaded at: https://www.mdpi.com/article/10.3390/cells15060498/s1, Supplementary Note S1: Power analysis for label-free proteomic quantification. Table S1: Details of the significantly identified proteins between the EC-Non obese and EC-Obese group; Table S2: Top scoring protein networks and associated functions identified by Ingenuity Pathway Analysis; Figure S1: Permutation test results for model validation illustrates the results of a 100-cycle permutation test used to assess the statistical significance and stability of the PLS-DA model; Supplementary Data S1: Summary of Ingenuity Pathway Analysis (IPA). References [66,67] are cited in the Supplementary Materials.
