# Supplementary material for: Proteomic Profiling of Endometrial Cancer Tissues Reveals Differential Expression of Proteomes in Obese Versus Non-Obese Patients
Source: Cells. 2026 Mar 11;15(6):498. doi: 10.3390/cells15060498 (PMC13024994; doi:10.3390/cells15060498)
Supplement: Supplementary file 1 [file cells-15-00498-s001.zip › Figure S1.pdf]

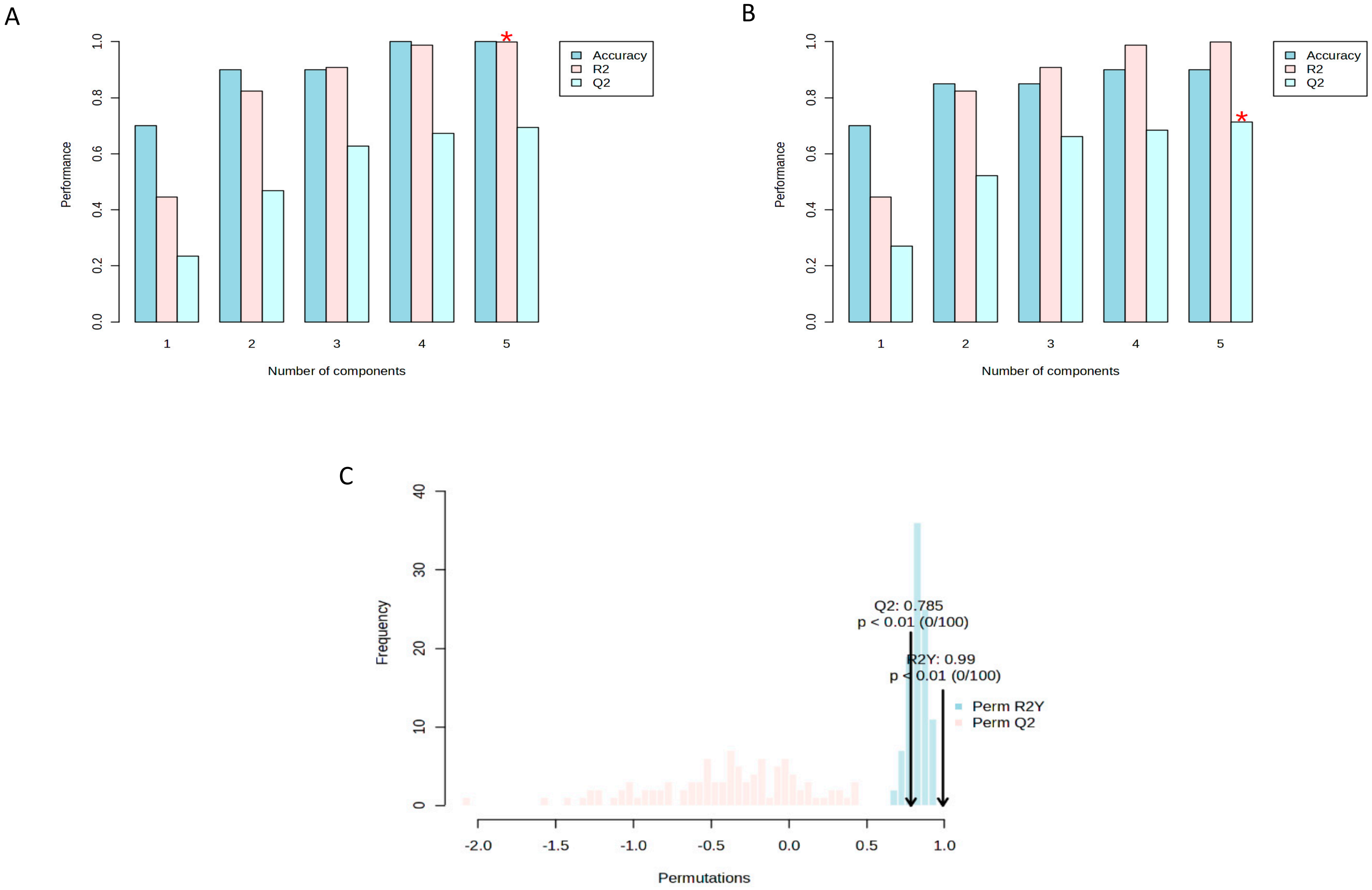

Figure S1. Permutation test results for model validation illustrates the results of a 100-cycle permutation test used to assess the statistical significance and stability of the PLS-DA model. (A, B) The histograms represent the null distribution of R2Y (light red bars) and Q2 (light blue bars) generated by randomly reassigning class labels. (C) The original model parameters are indicated by the vertical arrows, showing a goodness of fit (R2Y) of 0.99 and a predictive ability (Q2) of 0.785. The empirical p-values for both metrics were $p < 0.01$ (0/100), demonstrating that the original model significantly outperforms all permuted models. These results confirm that the observed separation between the EC Obese and EC Nonobese groups is statistically robust and not a product of random chance or overfitting.
