# Supplementary material for: Proteomic Profiling of Endometrial Cancer Tissues Reveals Differential Expression of Proteomes in Obese Versus Non-Obese Patients
Source: Cells. 2026 Mar 11;15(6):498. doi: 10.3390/cells15060498 (PMC13024994; doi:10.3390/cells15060498)
Supplement: Supplementary file 1 [file cells-15-00498-s001.zip › Supplementary Data 1 Summary.pdf]

INGENUITY
PATHWAY ANALYSIS

Analysis Name: IPA_EC_obese vs nonobese - 2025-07-14 12:11 PM
Analysis Creation Date: 2025-07-14
Build version: exported
Content version: 145030503 (Release Date: 2025-05-11)

Experiment Metadata

Name	Value

Analysis Settings

Reference set: Ingenuity Knowledge Base (Genes Only)
Relationship to include: Direct and Indirect
Does not Include Endogenous Chemicals
Optional Analyses: My Pathways My List

Filter Summary:
Consider only molecules and/or relationships where
(species = Human OR Uncategorized OR Mouse OR Rat) AND
(tissues/cell lines = Sciatic Nerve OR HCT-15 OR INS-1 OR Cartilage Tissue OR Osteosarcoma Cell Lines not otherwise specified OR Myeloma Cell Lines not otherwise specified OR H460 OR Liver OR Memory B cells OR Crypt OR Dermis OR A2780 OR Melanoma Cell Lines not otherwise specified OR Parietal Lobe OR Other T lymphocytes OR Effector memory RA+ cytotoxic T cells OR Substantia Nigra OR Breast Cancer Cell Lines not otherwise specified OR BDCA-1+ dendritic cells OR Myeloid dendritic cells OR Other Peripheral blood leukocytes OR

(c) 2000-2025 QIAGEN. All rights reserved.

PBMCs OR COLO205 OR MDA-MB-361 OR M14 OR Mammary Gland OR Osteoblasts OR P19 OR U2OS OR U937 OR THP-1 OR NCI-H226 OR CD4+ T-lymphocytes OR Thyroid Gland OR DU-145 OR Other Pancreatic Cancer Cell Lines OR KM-12 OR Other Immune cell lines OR CCRF-CEM OR T47-D OR Stem cells not otherwise specified OR Cortical neurons OR Gray Matter OR Bladder OR Vd1 Gamma-delta T cells OR Hep3B OR Trachea OR Adrenal Gland OR Jurkat OR Eosinophils OR HOP-62 OR OVCAR-5 OR Purkinje cells OR Langerhans cells OR LNCaP cells OR Testis OR Hippocampus OR Other Kidney Cancer Cell Lines OR Epithelial cells not otherwise specified OR NIH/3T3 cells OR Microvascular endothelial cells OR Other B lymphocytes OR Other Lymphocytes OR J774 OR Calvaria OR Oocytes OR U87MG OR HeLa OR NK cells not otherwise specified OR Th17 cells OR Kidney OR Pre-B lymphocytes OR Adipocytes OR SK-OV-3 OR Kidney cell lines not otherwise specified OR Macrophage Cancer Cell Lines not otherwise specified OR Cerebellum OR Megakaryocytes OR Cervical cancer cell line not otherwise specified OR Other CNS Cell Lines OR Natural T-regulatory cells OR Putamen OR SK-N-SH OR Stomach OR Pheochromocytoma cell lines not otherwise specified OR Other Immune cells OR Dendritic cells not otherwise specified OR Other Osteosarcoma Cell Lines OR Activated CD56bright NK cells OR SNB-75 OR RAW 264.7 OR Peripheral blood lymphocytes OR HS 578T OR BT-474 OR Thalamus OR Lymphoma Cell Lines not otherwise specified OR 3T3-L1 cells OR Other Ovarian Cancer Cell Lines OR Mature monocyte-derived dendritic cells OR Other Epithelial cells OR Monocyte-derived macrophage OR Adipose OR Immune cells not otherwise specified OR Neuroblastoma Cell Lines not otherwise specified OR Other Granulocytes OR NCI-H332M OR Endothelial cells not otherwise specified OR Plasmacytoid dendritic cells OR Hepatoma Cell Lines not otherwise specified OR TK-10 OR RXF-393 OR Mesenchymal stem cells OR Retina OR Subventricular Zone OR Other Prostate Cancer Cell Lines OR Dorsal Root Ganglion OR Lung Cancer Cell Lines not otherwise specified OR PC-12 cells OR Cells not otherwise specified OR NT2/D1 OR SK-MEL-5 OR Bone marrow-derived macrophages OR Vascular smooth muscle cells OR Other Stem cells OR Cos-7 cells OR Cerebral Cortex OR Smooth Muscle OR Other Kidney cell lines OR Spleen OR Other Organ Systems OR NCI-H23 OR Pituitary Gland OR Fibroblasts OR Cerebral Ventricles OR 786-0 OR Immune cell lines not otherwise specified OR Blood platelets OR Th1 cells OR RKO OR Other Macrophages OR Naive B cells OR Activated helper T cells OR Other Mononuclear leukocytes OR Effector memory cytotoxic T cells OR Microglia OR Min6 OR Other Monocytes OR Effector T cells OR Granulocytes not otherwise specified OR MDA-MB-231 OR CD34+ cells OR Pancreas OR CD56dim NK cells OR Thymus OR Epidermis OR Cell Line not otherwise specified OR Mast cells OR Pyramidal neurons OR UACC-257 OR Caudate Nucleus OR CAKI-1 OR Colon Cancer Cell Lines not otherwise specified OR Cornea OR Lymph node OR White Matter OR Large Intestine OR RBL-2H3 OR Placenta OR K-562 OR EKVX OR MOLT-4 OR PC-3 OR NB4 OR A549-ATCC OR A375 OR Corpus Callosum OR MCF7 OR HOP-92 OR LOX IMVI OR Trigeminal Ganglion OR Hematopoietic progenitor cells OR Murine NKT cells OR Chondrocytes OR Memory T lymphocytes not otherwise specified OR Nucleus Accumbens OR Olfactory Bulb OR Other Monocyte-derived dendritic cells OR UACC-62 OR Other Neuroblastoma Cell Lines OR Other Smooth muscle cells OR Forestomach OR Granule cells OR Skeletal Muscle OR Central memory cytotoxic T cells OR Ovarian Cancer Cell Lines not otherwise specified OR Other Teratocarcinoma Cell Lines OR RPMI-8266 OR Plasma cells OR CNS Cell Lines not otherwise specified OR MG-

(c) 2000-2025 QIAGEN. All rights reserved.

63 OR Granulosa cells OR Other Dendritic cells OR Bone marrow cells not otherwise specified OR Other Melanoma Cell Lines OR Other Memory T lymphocytes OR BA/F3 OR SF-268 OR Monocytes not otherwise specified OR Other Fibroblast cell lines OR NCI-ADR-RES OR 293 cells OR Caco2 cells OR Prostate Gland OR Striatum OR Sertoli cells OR MEF cells OR Effector memory helper T cells OR Fibroblast cell lines not otherwise specified OR Central memory helper T cells OR Nervous System not otherwise specified OR Neutrophils OR Vd2 Gamma-delta T cells OR HepG2 OR Other Endothelial cells OR Hypothalamus OR Stromal cells OR MDA-MB-468 OR MDA-MB-435 OR Esophagus OR SK-MEL-2 OR Other Neurons OR Thymocytes OR Peritoneal macrophages OR Splenocytes OR Keratinocytes OR HUVEC cells OR Lymphocytes not otherwise specified OR U266 OR Embryonic stem cells OR Lung OR Ovary OR Smooth muscle cells not otherwise specified OR Kidney Cancer Cell Lines not otherwise specified OR Neurons not otherwise specified OR Skin OR A498 OR SF-295 OR Hepatocytes OR Other Tissues and Primary Cells OR Organ Systems not otherwise specified OR Brain OR Activated CD56dim NK cells OR ACHN OR Other Cervical cancer cell line OR MDA-N OR Other Nervous System OR HCC-2998 OR Teratocarcinoma Cell Lines not otherwise specified OR Other Bone marrow cells OR OVCAR-3 OR BDCA-3+ dendritic cells OR Other Pheochromocytoma cell lines OR Ventricular Zone OR Intraepithelial T lymphocytes OR HT29 OR SW-620 OR Mononuclear leukocytes not otherwise specified OR Other Colon Cancer Cell Lines OR Other NK cells OR IGROV1 OR Bone marrow-derived dendritic cells OR SF-539 OR WEHI-231 OR Amygdala OR Immature monocyte-derived dendritic cells OR Spinal Cord OR Other Leukemia Cell Lines OR Other Breast Cancer Cell Lines OR Pro-B lymphocytes OR Macrophages not otherwise specified OR Leukemia Cell Lines not otherwise specified OR HCT-116 OR Other Macrophage Cancer Cell Lines OR OVCAR-8 OR Pancreatic Cancer Cell Lines not otherwise specified OR OVCAR-4 OR Melanocytes OR Other Cells OR Lens OR UO-31 OR HMC-1 OR Heart OR Beta islet cells OR Prostate Cancer Cell Lines not otherwise specified OR HL-60 OR Other Lymphoma Cell Lines OR HuH7 OR Naive helper T cells OR Small Intestine OR J-774A.1 OR U251 OR SK-MEL-28 OR Activated Vd1 Gamma-delta T cells OR Cardiomyocytes OR PANC-1 OR Brainstem OR Peripheral blood monocytes OR Other Hepatoma Cell Lines OR SW-480 OR Astrocytes OR Th2 cells OR Tissues and Primary Cells not otherwise specified OR Medulla Oblongata OR Choroid Plexus OR Granule Cell Layer OR Other Myeloma Cell Lines OR Activated Vd2 Gamma-delta T cells OR Other Lung Cancer Cell Lines OR Cytotoxic T cells OR BT-549 OR Salivary Gland OR CD56bright NK cells OR Swiss 3T3 cells OR T lymphocytes not otherwise specified OR MALME-3M OR HEL OR Other Cell Line OR SN12C OR Uterus OR SR OR B lymphocytes not otherwise specified OR Monocyte-derived dendritic cells not otherwise specified OR NCI-H522 OR Peripheral blood leukocytes not otherwise specified) AND

(data sources = An Open Access Database of Genome-wide Association Results OR BIND OR BioGRID OR Catalogue Of Somatic Mutations In Cancer (COSMIC) OR Chemical Carcinogenesis Research Information System (CCRIS) OR Clinical Genome Resource (ClinGen) OR ClinicalTrials.gov OR ClinVar OR Cognia OR DIP OR DrugBank OR Gene Ontology (GO) OR GVK Biosciences OR Hazardous Substances Data Bank (HSDB) OR HumanCyc OR Ingenuity Expert Findings OR Ingenuity ExpertAssist Findings OR IntAct OR Interactome studies OR MIPS OR miRBase OR miRecords OR Mouse Genome Database (MGD) OR Obesity Gene Map Database OR Online Mendelian Inheritance in

(c) 2000-2025 QIAGEN. All rights reserved.

Man (OMIM) OR Reactome OR TarBase OR TargetScan Human OR TargetScan Mouse)

Top Canonical Pathways

Name	p-value	Overlap
**Neutrophil degranulation**	9.46E-15	7.8 % 37/477
**Cell surface interactions at the vascular wall**	2.11E-13	11.2 % 24/214
**Binding and Uptake of Ligands by Scavenger Receptors**	7.53E-11	14.0 % 16/114
**Smooth Muscle Contraction**	8.41E-11	25.6 % 11/43
**Extracellular matrix organization**	2.98E-09	13.1 % 14/107

Top Upstream Regulators

Upstream Regulators

Name	p-value	Predicted Activation
**TGFB1**	1.93E-22	Activated
**SRF**	1.32E-18	Activated
**KRAS**	1.95E-18	
**dexamethasone**	9.80E-18	
**YAP1**	3.11E-15	

(c) 2000-2025 QIAGEN. All rights reserved.

INGENUITY PATHWAY ANALYSIS

Causal Network

Name	p-value	Predicted Activation
**HJURP**	5.56E-28	
**F2R**	1.25E-26	
**rosmarinic acid**	2.26E-26	Inhibited
**Aminoacyl-tRNA Synthetase**	2.39E-26	
**MRTFA**	3.54E-26	Activated

Top Diseases and Bio Functions

Diseases and Disorders

Name	p-value range	# Molecules
**Cancer**	1.45E-04 - 3.17E-34	428
**Organismal Injury and Abnormalities**	1.45E-04 - 3.17E-34	436
**Endocrine System Disorders**	1.45E-04 - 6.79E-26	394
**Cardiovascular Disease**	1.23E-04 - 1.91E-24	244
**Neurological Disease**	1.35E-04 - 1.91E-24	353

Molecular and Cellular Functions

Name	p-value range	# Molecules
**Cellular Movement**	1.11E-04 - 7.32E-18	185

(c) 2000-2025 QIAGEN. All rights reserved.

INGENUITY PATHWAY ANALYSIS

Cellular Assembly and Organization	5.08E-05 - 1.10E-15	165
Cellular Function and Maintenance	1.25E-04 - 1.10E-15	238
Cell Morphology	1.44E-04 - 7.22E-15	147
Cell Death and Survival	1.22E-04 - 1.34E-13	205

Physiological System Development and Function

Name	p-value range	# Molecules
Organismal Survival	8.87E-05 - 2.74E-22	199
Tissue Morphology	1.16E-04 - 6.32E-14	153
Organismal Development	1.39E-04 - 1.53E-12	188
Tissue Development	1.39E-04 - 1.53E-12	173
Cardiovascular System Development and Function	1.35E-04 - 1.55E-11	114

Top Tox Functions

Assays: Clinical Chemistry and Hematology

Name	p-value range	# Molecules
Increased Levels of Potassium	3.54E-02 - 1.33E-02	3
Increased Levels of Creatinine	1.22E-01 - 2.83E-02	3
Decreased Levels of Albumin	1.43E-01 - 6.48E-02	2
Increased Levels of Hematocrit	9.80E-02 - 9.80E-02	4
Increased Levels of Red Blood Cells	1.49E-01 - 1.49E-01	4

(c) 2000-2025 QIAGEN. All rights reserved.

INGENUITY PATHWAY ANALYSIS

Cardiotoxicity

Name	p-value range	# Molecules
Cardiac Dysfunction	4.44E-01 - 1.18E-22	59
Cardiac Dilation	3.64E-01 - 1.40E-11	36
Cardiac Enlargement	2.26E-01 - 1.40E-11	47
Heart Failure	4.44E-01 - 1.23E-09	33
Cardiac Arrythmia	1.00E00 - 2.16E-09	37

Hepatotoxicity

Name	p-value range	# Molecules
Liver Hyperplasia/Hyperproliferation	5.22E-01 - 1.25E-14	234
Hepatocellular carcinoma	5.22E-01 - 3.48E-09	106
Liver Inflammation/Hepatitis	4.97E-01 - 8.81E-05	23
Liver Fibrosis	3.53E-01 - 3.09E-04	29
Liver Necrosis/Cell Death	3.62E-01 - 3.60E-04	15

Nephrotoxicity

Name	p-value range	# Molecules
Renal Enlargement	3.30E-02 - 6.66E-07	10
Kidney Failure	4.08E-01 - 8.78E-06	19

(c) 2000-2025 QIAGEN. All rights reserved.

INGENUITY PATHWAY ANALYSIS

**Renal Necrosis/Cell Death**	4.94E-01 - 7.89E-05	22
**Renal Proliferation**	9.56E-02 - 1.01E-04	16
**Glomerular Injury**	5.06E-01 - 2.75E-04	32

Top Regulator Effect Networks

ID	Regulators	Disease & Functions	Consistency Score
**1**	DNMT3A,HOXA10,IKZF1,IL1 (family),IL17A (+1 more)	Engulfment of antigen presenting cells (+2 more)	11.667
**2**	ANGPT2,CASC2,CNR1,ERBB4,GPER1,HNF4A,INSIG 1,mir-802 (+5 more)	Dilated or arrhythmogenic cardiomyopathy (+3 more)	10.634
**3**	ATF4,HELLS (+3 more)	Cell proliferation of tumor cell lines (+3 more)	9.899
**4**	HNF4A,miR-3081-3p (miRNAs w/seed UGCGCUC) (+7 more)	Abnormal morphology of urinary system (+5 more)	7.546
**5**	ATF4,SCAP,SKIC2	Cell proliferation of tumor cell lines (+5 more)	7.211

Top Networks

ID	Associated Network Functions	Score
**1**	Amino Acid Metabolism, Post-Translational Modification, Small Molecule Biochemistry	103
**2**	Cell Morphology, Cellular Movement, Connective Tissue Development and Function	82

(c) 2000-2025 QIAGEN. All rights reserved.

INGENUITY PATHWAY ANALYSIS

**3**	Cellular Movement, Metabolic Disease, Organismal Injury and Abnormalities	53
**4**	Cancer, Organismal Injury and Abnormalities, Nervous System Development and Function	51
**5**	Connective Tissue Disorders, Hematological Disease, Hereditary Disorder	49

Top Tox Lists

Name	p-value	Overlap
**LXR/RXR Activation**	2.78E-07	10.0 % 13/130
**Hepatic Fibrosis**	5.09E-05	4.4 % 21/473
**Positive Acute Phase Response Proteins**	1.25E-04	16.7 % 5/30
**Mitochondrial Dysfunction**	3.41E-04	4.5 % 16/356
**Genes associated with Chronic Allograft Nephropathy (Human)**	3.60E-04	19.0 % 4/21

Top My Lists

Top My Pathways

(c) 2000-2025 QIAGEN. All rights reserved.

INGENUITY PATHWAY ANALYSIS

Name	p-value	Overlap
**Network 2**	2.30E-02	6.2 % 4/65

Top ML Disease Pathways

Name	p-value	Overlap
**Lipodystrophy**	2.66E-04	14.3 % 5/35
**Urea cycle disorder**	2.66E-04	14.3 % 5/35
**Autoimmune anemia**	2.66E-04	14.3 % 5/35
**Aneurysm**	3.48E-04	13.5 % 5/37
**Hyperammonemia**	3.48E-04	13.5 % 5/37

Top Analysis-Ready Molecules

Expr Fold Change

Molecules	Expr. Value	Chart
**PICK1**	↑ 4.365	
**TRAM1**	↑ 4.175	
**TATDN3**	↑ 4.047	
**TRIM5**	↑ 3.274	
**MAPK8IP3**	↑ 3.158	
**VAT1L**	↑ 3.147	

(c) 2000-2025 QIAGEN. All rights reserved.

INGENUITY PATHWAY ANALYSIS

**VPS37C**	↑ 3.097	
**RPL37**	↑ 2.930	
**ARFGAP2**	↑ 2.902	
**MYL4**	↑ 2.878	

Expr Fold Change

Molecules	Expr. Value	Chart
**TCAF1**	↓ -9.615	
**SBSN**	↓ -8.772	
**LDAH**	↓ -8.264	
**DERL3**	↓ -8.065	
**CEMIP2**	↓ -6.944	
**ITPR2**	↓ -6.897	
**DGAT1**	↓ -6.849	
**TMEM50B**	↓ -6.623	
**ISCA2**	↓ -6.494	
**MAN1B1**	↓ -6.410	

(c) 2000-2025 QIAGEN. All rights reserved.

INGENUITY PATHWAY ANALYSIS
