# Supplementary material for: Proteomic Profiling of Endometrial Cancer Tissues Reveals Differential Expression of Proteomes in Obese Versus Non-Obese Patients
Source: Cells. 2026 Mar 11;15(6):498. doi: 10.3390/cells15060498 (PMC13024994; doi:10.3390/cells15060498)
Supplement: Supplementary file 1 [file cells-15-00498-s001.zip › Supplementary note 1.pdf]

Power Analysis for Label-Free Proteomic Quantification

Power analysis was conducted using a two-sample t-test framework on log2-transformed protein intensities, consistent with linear mixed-effects modeling approaches implemented in MSstats [66]. Assuming a standard deviation of 0.4 (Multi-laboratory benchmarking and large-scale proteomic reproducibility studies have consistently reported median protein-level coefficients of variation (CVs) in the range of ~20–40% following normalization, depending on platform and experimental design (e.g., $\alpha = 0.05$, a sample size of 10 per group provides 80% power to detect ≥1.5-fold protein expression changes [67]. This estimate accounts for expected proteomic variability observed in label-free quantification.

66. Choi M, Chang CY, Clough T, Broudy D, Killeen T, MacLean B, Vitek O. MSstats: an R package for statistical analysis of quantitative mass spectrometry-based proteomic experiments. Bioinformatics. 2014 Sep 1;30(17):2524-6. doi: 10.1093/bioinformatics/btu305.

67. Tian S, Zhan D, Yu Y, Wang Y, Liu M, Tan S, Li Y, Song L, Qin Z, Li X, Liu Y, Li Y, Ji S, Wang S; Proteomic Massive Analysis and Quality Control Consortium; Zheng Y, He F, Qin J, Ding C. Quartet protein reference materials and datasets for multi-platform assessment of label-free proteomics. Genome Biol. 2023 Sep 7;24(1):202. doi: 10.1186/s13059-023-03048-y.
